# Case Report: Hematopoietic stem cell transplantation in an adult patient with X-linked agammaglobulinemia and severe refractory enteropathy

**DOI:** 10.3389/fimmu.2025.1662016

**Published:** 2025-09-02

**Authors:** Paula Teresa López-León, Marta Dafne Cabañero-Navalon, Victor Garcia-Bustos, Francisco Giner, Héctor Balastegui Martín, Pedro Asensi Cantó, Juan Montoro Gómez, Olga Seguí-Cotano, María Argente Pla, Pedro Moral Moral

**Affiliations:** ^1^ Primary Immunodeficiencies Unit, Department of Internal Medicine, La Fe University and Polytechnic Hospital, Valencia, Spain; ^2^ Research Group of Chronic Diseases and HIV Infection, Health Research Institute La Fe, Valencia, Spain; ^3^ Unit of Infectious Diseases, La Fe University and Polytechnic Hospital, Valencia, Spain; ^4^ Severe Infection Research Group, Health Research Institute La Fe, Valencia, Spain; ^5^ Department of Pathology, La Fe University and Polytechnic Hospital, Valencia, Spain; ^6^ Hematology Department, La Fe University and Polytechnic Hospital, Valencia, Spain; ^7^ Hematology Research Group, Health Research Institute La Fe, Valencia, Spain; ^8^ School of Medicine and Dentistry, Catholic University of Valencia, Valencia, Spain; ^9^ Endocrinology and Nutrition Department, La Fe University and Polytechnic Hospital, Valencia, Spain; ^10^ Joint Research Unit on Endocrinology, Nutrition and Clinical Dietetics, Health Research Institute La Fe, Valencia, Spain

**Keywords:** x-linked agammaglobulinemia, hematopoietic stem cell transplantation, primary immunodeficiency, chronic enteropathy, norovirus infection

## Abstract

X-linked agammaglobulinemia (XLA) is a rare primary immunodeficiency characterized by absent B cells and severe hypogammaglobulinemia. While lifelong immunoglobulin replacement therapy (IgRT) effectively prevents severe infections, it does not prevent chronic complications in a subset of patients with immune dysregulation. We report the case of a young adult with genetically confirmed XLA and severe, treatment-refractory enteropathy with persistent *Campylobacter jejuni* and norovirus infections, who underwent successful allogeneic hematopoietic stem cell transplantation (HSCT) after exhausting all therapeutic options. Post-transplant, the patient achieved complete resolution of chronic diarrhea, clearance of enteric pathogens, and sustained independence from parenteral nutrition, with significant improvement in nutritional status, bone density, and quality of life. This case represents one of the few documented adult XLA transplants and highlights HSCT as a feasible, safe, and potentially curative option in selected patients with severe non-hematologic complications. It underscores the need to consider HSCT earlier in the disease course, especially when organ damage is progressive and irreversible. Further studies are needed to clarify indications, timing, and cost-effectiveness of HSCT in XLA.

## 1 Introduction

X-linked agammaglobulinemia (XLA) is a rare primary immunodeficiency (PID) caused by pathogenic variants in the Bruton’s tyrosine kinase (*BTK*) gene, resulting in a profound defect in B cell maturation and subsequent agammaglobulinemia (1). While the clinical phenotype is dominated by recurrent bacterial infections due to humoral immunodeficiency, BTK expression in myeloid and dendritic cell lineages also implicates this kinase in broader immune regulation, including Toll-like receptor signaling and innate antiviral responses (2). Furthermore, the absence of B cell – T cell interaction compromises T-cell homeostasis and adaptive immunity (3).

Lifelong immunoglobulin replacement therapy (IgRT) remains the cornerstone of management in XLA, effectively preventing most severe infections. However, IgRT provides only IgG, leaving persistent deficiencies in IgA and IgM—critical for mucosal immunity (4). It also fails to restore BTK expression in non–B-cell compartments, including dendritic cells and myeloid subsets. These immunological gaps are believed to underlie the development of a “complicated phenotype” in a subset of patients, manifested by chronic organ damage, recurrent infections, increased malignancy risk, inflammatory complications, and reduced quality of life (5).

Among these, chronic gastrointestinal disease—often associated with enteropathogens such as *Campylobacter* spp. or norovirus—is increasingly recognized as a severe and underappreciated manifestation, occasionally culminating in intestinal failure (6, 7). Norovirus persistence, in particular, has been implicated in progressive mucosal injury and villous atrophy which may be driven not only by immune-mediated mechanisms, but also by direct viral cytotoxicity, especially in patients with antibody deficiencies, for whom viral clearance mechanisms are impaired (8).

Current therapeutic strategies for chronic viral enteropathy in PID remain largely empirical and frequently ineffective. Experimental interventions—including oral immunoglobulins, ribavirin, nitazoxanide, and fecal microbiota transplantation (FMT) — have yielded inconsistent outcomes (9–11). In selected cases of humoral immunodeficiency with refractory disease and life-threatening complications, allogeneic hematopoietic stem cell transplantation (HSCT) has been investigated as a potential curative strategy. However, its role in XLA remains controversial and infrequently reported, predominantly in high-risk cases, particularly during infancy (12, 13).

We report the case of an adult patient with genetically confirmed XLA and chronic intestinal failure with persistent norovirus and recurrent *Campylobacter jejuni* infections, who underwent successful allogeneic HSCT after exhausting all available therapeutic options. This case illustrates the immunological, clinical, and functional benefits of HSCT in a patient with XLA and a complex gastrointestinal phenotype, and raises the question of whether earlier transplantation should be considered in similar severe cases of immune dysregulation.

## 2 Case report

### 2.1 Patient information

An 18-year-old male with a confirmed diagnosis of X-linked agammaglobulinemia was referred to adult PID Unit for evaluation due to progressive gastrointestinal failure, recurrent systemic infections, and treatment-refractory osteoporosis. The diagnosis of XLA had been established at 6 months of age following an episode of bilateral pneumonia, with subsequent genetic testing identifying a hemizygous splice site variant in the *BTK* gene: c.895-2del (NM_000061.3), affecting the canonical splice acceptor site of intron 10.

**Figure 1:**
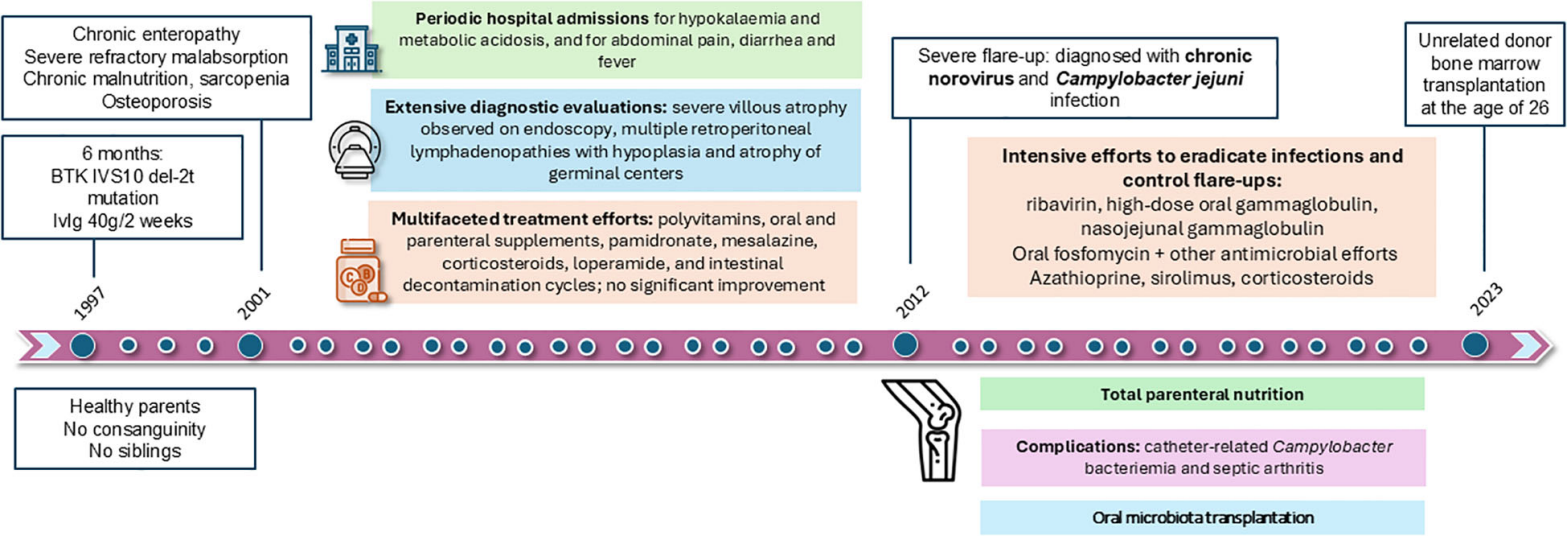
Clinical timeline from diagnosis of XLA to hematopoietic stem cell transplantation. Chronological summary of major clinical events, diagnostic findings, and therapeutic interventions in our patient with XLA.

Since infancy, the patient had been treated with high-dose intravenous immunoglobulin replacement therapy (IgRT), initially 40 g every two weeks and subsequently increased to 50 g every two weeks during adolescence, due to suboptimal infectious control and intolerance to subcutaneous administration (SCIg) related to high infusion volumes. These unusually elevated doses were necessary to compensate for extensive gastrointestinal immunoglobulin losses associated with severe enteropathy. Despite this intensified regimen, the patient continued to experience recurrent sinopulmonary infections, profound malabsorption, and progressive osteoporosis. Following allogeneic HSCT at age 26 (**Figure 1**), sustained clinical improvement enabled progressive tapering of IgRT, which is currently maintained at 20 g every three weeks.

### 2.2 Family history

The patient was born to non-consanguineous parents with a significant maternal family history of XLA. His mother is a known carrier of the IVS10 del-2 mutation, as are four of his maternal aunts and one female maternal cousin. The patient’s maternal family history includes multiple early childhood deaths of male relatives, suggestive of undiagnosed XLA-related complications. His maternal grandmother had two sons who died in early infancy, one at 4 months and another at 5 years of age. Additionally, a maternal aunt had a son who passed away at 18 months of age. The patient’s father had congenital ichthyosis, but no known immunodeficiency. No other chronic illnesses or immunodeficiencies were reported in the family.

### 2.3 Clinical course and multisystemic involvement

During the first years of life, despite intravenous IgRT, the patient experienced recurrent upper respiratory infections and diarrhea, leading to multiple emergency visits and antibiotic courses. He exhibited severe failure to thrive, with weight and height consistently below the 3rd percentile.

**Figure 2:**
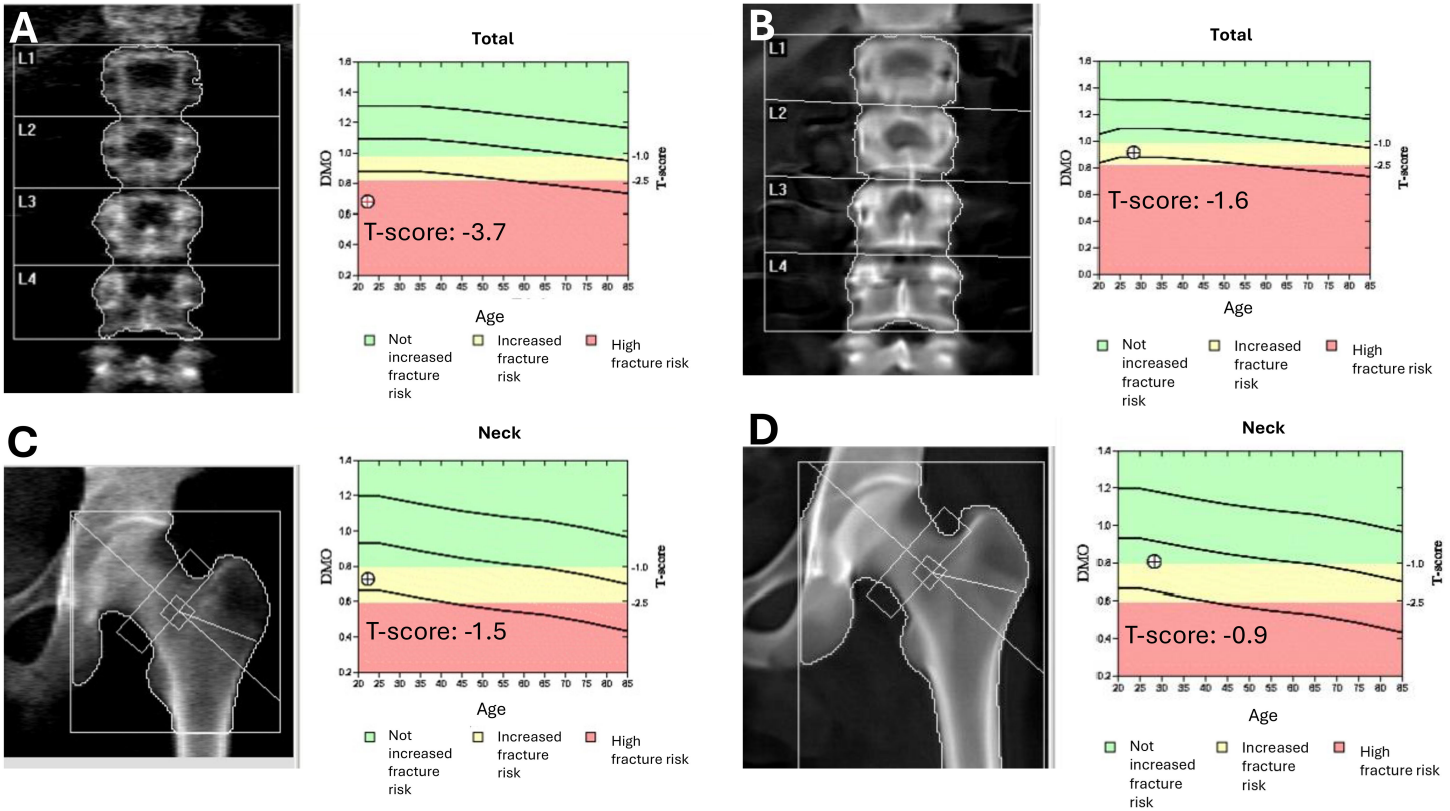
Evolution of bone mineral density before and after hematopoietic stem cell transplantation. DXA scans of the lumbar spine **(A, B)** and femoral neck **(C, D)** before **(A, C)** and 14 months after **(B, D)** allogeneic hematopoietic stem cell transplantation (HSCT). Pre-transplant scans revealed reduced bone mineral density [T-scores: –3.7 at the lumbar spine **(A)** and –1.5 at the femoral neck **(C)**]. Post-transplant assessments showed significant improvement [T-scores: –1.6 **(B)** and –0.9, respectively **(D)**], indicating partial recovery of bone health following HSCT and the resolution of chronic gastrointestinal dysfunction.

At 4 years of age, the patient developed chronic diarrhea, malabsorption and iron deficiency, requiring follow-up with Pediatric Endocrinology and Gastroenterology. Initial duodenoscopy and colonoscopy revealed lymphocytic duodenitis with absence of plasma cells in the duodenal and colonic mucosa. Despite multiple cyclic antibiotic therapies symptoms persisted throughout childhood. By 14 years of age, a repeated endoscopy showed disease progression, including stage II esophagitis, chronic gastritis with fibrosis, focal villous atrophy, and colonic fibrosis. Gluten-free diet was trialed despite negative celiac serology but resulted in minimal improvement. Treatment with salicylates and enteric corticosteroids led to partial improvement of diarrhea and were maintained; however, growth failure persisted despite an unrestrictedbalanced diet providing approximately 3,500 kcal/day. The patient’s weight remained below the 10th percentile, with a BMI of 16 kg/m², consistent with undernutrition, and early-onset osteoporosis (Z-score < -2.4) was diagnosed at age 13 requiring pamidronate treatment (**Figures 2A, C**).

At 16 years of age, a capsule endoscopy showed severe gastrointestinal atrophy, leading to attempts at oral nutritional supplementation and nighttime enteral nutrition via a nasogastric tube, which was not tolerated. Home parenteral nutrition (HPN) five nights per week was initiated at 16 years of age leading to partial progressive improvement in weight but persistent diarrhea, metabolic complications, and delayed bone age persisted.

**Figure 3:**
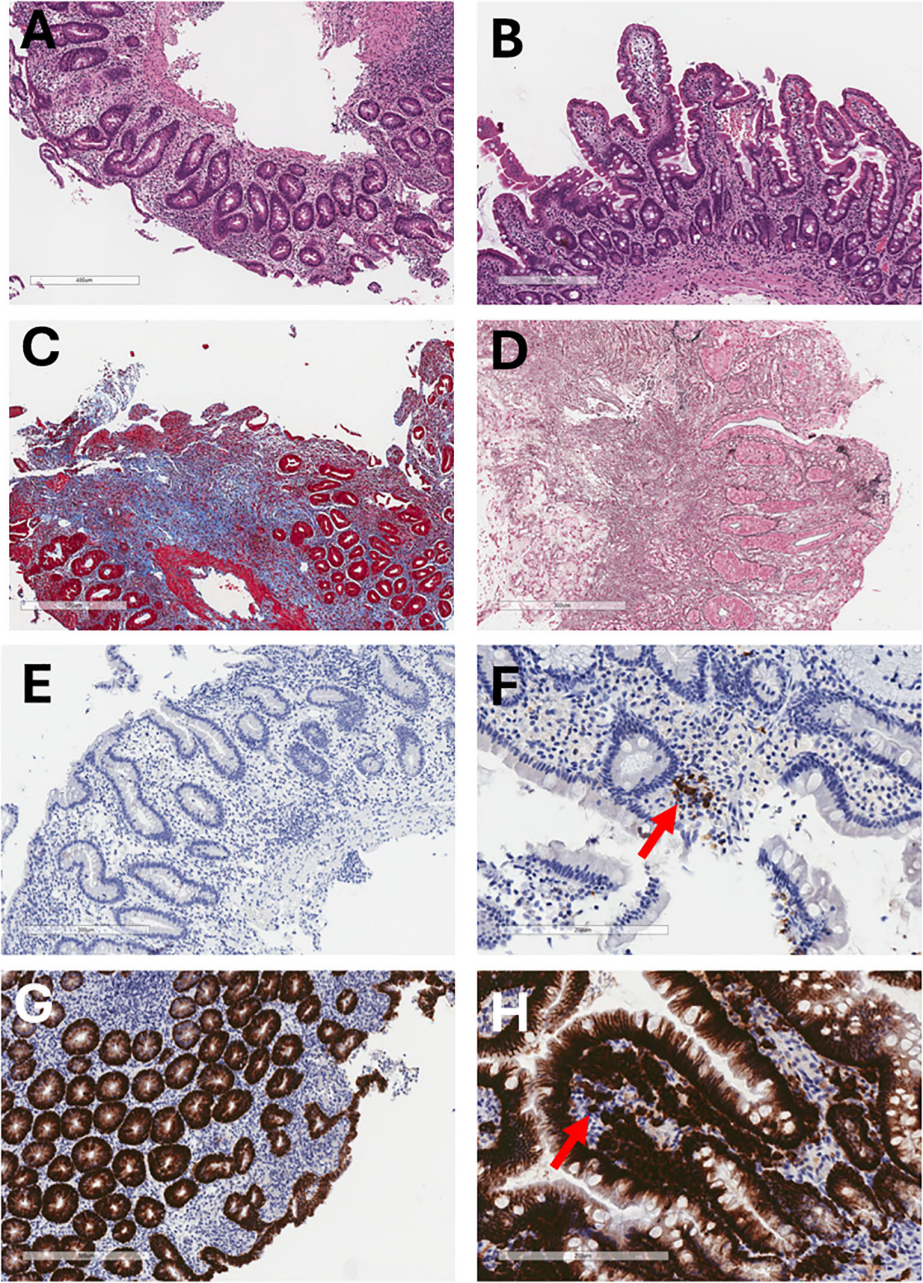
Histological and immunohistochemical evolution of the duodenal mucosa before and after allogeneic HSCT. **(A)** Pre-HSCT hematoxylin and eosin staining shows chronic duodenitis with superficial epithelial erosion and complete villous atrophy (6.6×). **(B)** One year post-HSCT, hematoxylin and eosin staining shows restoration of normal villous architecture without chronic inflammation (8×). **(C)** Pre-HSCT Masson’s trichrome staining demonstrates marked collagen type I deposition in the lamina propria, indicative of severe fibrosis. **(D)** Pre-HSCT Wilder’s reticulin stain highlights prominent reticular fiber deposition (black) in the lamina propria. **(E)** Pre-HSCT CD20 immunostaining shows absence of B lymphocytes in the lamina propria and marked villous atrophy (10×). **(F)** One year post-HSCT, CD20 immunostaining shows scattered B lymphocytes in the lamina propria (arrow) (20×). **(G)** Pre-HSCT CD138 immunostaining reveals complete absence of plasma cells in the lamina propria (10×). **(H)** One year post-HSCT, CD138 staining demonstrates abundant plasma cells (arrow) and preserved villous structure (20×).

Between the ages of 18 and 20, the patient required multiple hospital admissions due to metabolic acidosis and hypokalemia, which were managed with intravenous fluids, bicarbonate, and antibiotics. Repeated capsule endoscopy and enteroscopy confirmed persistent severe villous atrophy (**Figure 3A**), fibrin-covered aphthae, fibrosis (**Figures 3C, D**), (absence of B cells (**Figure 3E**) and plasma cells (**Figure 3G**), in spite of chronic treatment with topical corticosteroids and cholestyramine resin.

At 21 years of age, the patient was admitted to hospital with a Port-a-Cath–associated bloodstream infection caused by *Staphylococcus epidermidis* and *Campylobacter jejuni*, the latter identified for the first time. During the same admission, he developed *C. jejuni* septic arthritis and osteomyelitis of the knee. Both infections were attributed to bacterial translocation secondary to underlying gastrointestinal colonization. Simultaneously, norovirus was detected for the first time by PCR in both stool and ileal biopsy samples. Following multidisciplinary consultation with the Infectious Diseases team, a therapeutic trial with oral ribavirin and enteral immunoglobulins—initially administered orally alongside high-dose proton pump inhibitors and subsequently via nasojejunal tube—was initiated. However, the regimen was discontinued shortly thereafter due to worsening diarrhea. Treatment was switched to sirolimus, targeting norovirus and enteropathy, resulting in modest improvement in stool frequency. At age 22, teduglutide—a glucagon-like peptide-2 analogue that promotes intestinal mucosal growth—was introduced. Although initial weight gain was observed, the therapy was discontinued after 8 months due to lack of sustained efficacy. Moreover, regardless pamidronate treatment, osteoporosis progressed, and it was replaced by denosumab at age of 23, resulting in severe hypocalcemia requiring hospitalization.

At 25 years of age, following four years of recurrent episodes, the patient had required seven additional hospital admissions for severe diarrhea, with *C. jejuni* and norovirus detected in nearly every episode. After the initial isolation of *C. jejuni*, prophylactic azithromycin was initiated, and the patient subsequently received multiple inpatient courses of intravenous carbapenems (meropenem and ertapenem), in addition to prolonged outpatient regimens of oral fosfomycin (lasting 4–6 weeks). These treatments led to transient negativization of stool cultures and PCR, though relapses consistently occurred within a few months. Antimicrobial susceptibility testing revealed an initial C. jejuni strain sensitive to macrolides but resistant to quinolones and tetracyclines; the final isolate before HSCT showed acquired resistance to meropenem. In an attempt to modulate the intestinal microbiota, FMT was performed following a course of *C. jejuni* eradication therapy with fosfomycin. The patient received an oral formulation of lyophilized fecal microbiota (MBK-01), administered as a single dose of four 250 mg capsules, which led to a marked exacerbation of diarrhea with sepsis, and metabolic acidosis, necessitating a two-month hospital stay. However, no specific microbial etiology was isolated.

### 2.4 Hematopoietic stem cell transplantation and outcomes

At 26 years of age, in May 2023, due to chronic malabsorption, recurrent *C. jejuni* infections, norovirus persistence, and failure of multiple eradication regimens, the patient was considered for allogeneic HSCT. After myeloablative conditioning consisting of thiotepa (5 mg/kg, on days -7 and -6), intravenous busulfan (3.2 mg/kg, on days -5 and -4), and fludarabine (50 mg/kg, on days -5, -4, and -3), he received an unmanipulated bone marrow graft from a fully HLA-matched (10/10) unrelated donor. Graft-versus-host disease (GVHD) prophylaxis included post-transplant cyclophosphamide (50 mg/kg, on days +3 and +4), along with sirolimus and mycophenolate mofetil starting on day +5. Neutrophil engraftment was achieved by day +18 and the patient was discharged without complications. Platelet engraftment occurred on day +31.

Following HSCT in May 2023, the patient exhibited a marked clinical improvement across multiple domains, particularly in gastrointestinal function, infectious burden, and overall nutritional status. One of the most significant post-transplant outcomes was the gradual resolution of chronic diarrhea and malabsorption syndrome. Within one-month post-HSCT, *C. jejuni* was undetectable in faecal samples, and norovirus was cleared by the second month post-transplantation, a remarkable achievement considering its persistent detection over the preceding years.

Endoscopic re-evaluation performed at 14 months post-HSCT revealed only minimal residual villous atrophy in the duodenum, in contrast to the severe atrophic changes observed pre-transplantation. The colonic mucosa appeared entirely normal—indicating resolution of the colonic fibrosis described in pre-HSCT evaluations—and histopathological analysis of duodenal biopsies demonstrated reconstitution of normal mucosal architecture (**Figure 3B**). Only minor inflammatory changes were observed in the colonic tissue, and, notably, B cells and plasma cells were present, confirming immune reconstitution at the mucosal level (**Figures 3F, H**, respectively). These findings were further supported by fecal biomarkers: alpha-1 antitrypsin (A1AT), which had been consistently elevated prior to HSCT (up to 3.2 mg/g; normal <1.5 mg/g), normalized after transplantation (0.8–1.3 mg/g in follow-up assessments), indicating resolution of enteric protein loss. Similarly, fecal calprotectin levels decreased markedly from a pre-transplant mean of 830 µg/g to 150 µg/g post-HSCT, suggesting a significant reduction in mucosal inflammation.

**Figure 4:**
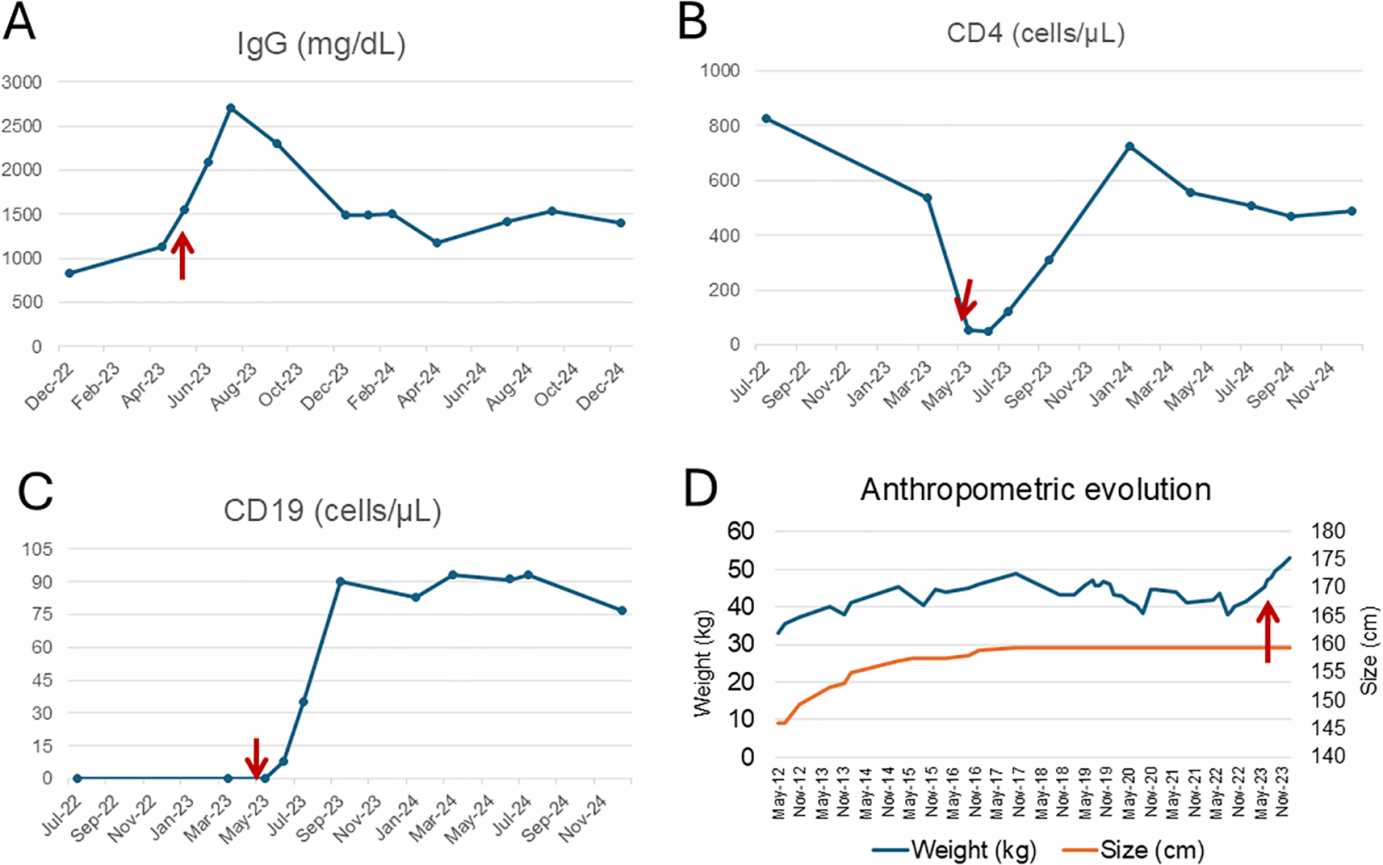
Immunological and anthropometric evolution before and after hematopoietic stem cell transplantation. Longitudinal evolution of IgG levels **(A)**, CD4^+^ T cells **(B)**, CD19^+^ B cells **(C)**, and anthropometric parameters **(D)** in a patient with XLA. The red arrows indicate the time of allogeneic HSCT (May 2023). **(A)** shows a marked post-transplant increase in IgG levels, followed by the discontinuation of intravenous IgRT. However, due to subsequent *Streptococcus pneumoniae* infections, low-dose IgRT was reintroduced. **(B)** illustrates CD4^+^ T cell nadir prior to HSCT with progressive recovery thereafter. **(C)** reveals a sustained increase in peripheral B cells (CD19^+^), primarily reflecting pre-germinal center subsets, consistent with partial reconstitution. **(D)** demonstrates anthropometric changes over time. While height increased gradually following initiation of home parenteral nutrition (HPN), it remained below the age-appropriate percentile. In contrast, weight significantly improved after HSCT, coinciding with resolution of enteropathy and nutritional autonomy.

Due to this significant gastrointestinal improvement, the patient was able to progressively transition from HPN to full oral intake, a milestone that had not been accomplished despite over a decade of nutritional support. By the end of 2023, HPN was successfully discontinued, and the Port-a-Cath was removed, marking a major improvement in the patient’s quality of life. Notably, following HSCT, the patient experienced a substantial weight gain—from under 40 kg pre-transplant to 53 kg—clearly attributable to restored gastrointestinal function and effective nutritional absorption enabled by the transplant (**Figure 4D**).

Post-transplant bone mineral density showed notable improvement. A dual-energy X-ray absorptiometry (DXA) scan performed in February 2025 demonstrated a T-score of -1.6 and Z-score of -1.6 at the lumbar spine, and a T-score of -0.9 and Z-score of -0.8 at the femoral neck. These values represent substantial improvement compared to previous assessments, where severe osteoporosis had been present (**Figures 2B, D**).

At month +8 post-HSCT, the patient developed asymptomatic lichen planus–like lesions of the oral mucosa, papulosquamous skin eruptions involving approximately 9% of body surface area, and keratoconjunctivitis sicca. These findings were consistent with mild chronic GVHD. Management included topical corticosteroids and topical tacrolimus for mucocutaneous involvement, and autologous serum eye drops for ocular symptoms. Sirolimus, originally initiated prior to HSCT for refractory immune-mediated enteropathy, was continued post-transplant as part of GVHD prophylaxis. By the most recent follow-up at month +24, there were no clinical signs of active GVHD, and a tapering regimen of sirolimus had been initiated, with complete discontinuation planned by month +28. Sirolimus trough levels remained within the therapeutic range throughout the post-transplant period (mean: 8.7 ng/mL).

Although the patient remained susceptible to mild upper respiratory tract infections, their frequency and severity markedly decreased following HSCT. He experienced three episodes of *Streptococcus pneumoniae* pneumonia during the first year post-transplant, all of which required hospitalization and intravenous antibiotics, with full clinical recovery and no long-term complications. IgRT, initially tapered and discontinued in January 2024, was subsequently reintroduced at a reduced dose (20 g every three weeks), which is currently maintained. Immunophenotypic monitoring +20 months after transplantation showed progressive B-cell reconstitution, characterized by an expansion of CD19^+^ naïve B cells (CD27^−^IgD^++^IgM^++^) to 87 cells/uL but a limited population of post-germinal center (CD27+) of 11 cells/uL with a class-switched memory B cells count of 5 cells/uL. Total T-cell counts also increased to normal range. Serum immunoglobulin levels demonstrated gradual improvement: IgA rose from <5 mg/dL pre-transplant to 65 mg/dL at 26 months post-transplant, and IgM from <5 mg/dL to 50 mg/dL (**Figures 4A–C**). In May 2025, the patient received a *Salmonella typhi* Vi polysaccharide vaccine as part of immune monitoring. The post-vaccination response was partial, reflecting ongoing but incomplete recovery of humoral function.

The clinical improvements achieved following allogeneic HSCT have had a profound impact on the patient’s quality of life. The resolution of chronic diarrhea and malabsorption enabled the discontinuation of HPN and the removal of the Port-a-Cath, allowing for greater physical autonomy and freedom from hospital-based care. Nutritional status, bone density, and overall functional capacity improved markedly, facilitating a gradual return to daily activities and social engagement. Although mild infectious susceptibility and limited chronic GVHD persist, the post-transplant course has been transformative, enabling the patient to reclaim a degree of normalcy that had been previously unattainable.

## 3 Discussion

This case represents one of the few documented cases of successful allogeneic HSCT in an adult patient with XLA and severe treatment-refractory enteropathy. In our patient, the resolution of chronic diarrhea, clearance of persistent *C. jejuni* and norovirus infections, and sustained independence from HPN underscore the transformative impact of HSCT in this setting. This case adds to emerging evidence that HSCT can restore immune function in XLA (5, 14–16) and underscores its potential as a curative option in patients with severe, treatment-refractory complications, although not being routinely indicated. It also prompts consideration of earlier HSCT in selected patients with progressive immune dysregulation.

Chronic gastrointestinal involvement in XLA and other humoral immunodeficiencies, though less commonly described than respiratory or lymphoid complications, is increasingly recognized as a source of substantial morbidity (5). In some patients, additional mutations in genes such as *CYBB* have been identified, contributing to atypical phenotypes characterized by immune dysregulation and severe enteropathy (17). Mechanistic insights from previous studies have also highlighted impaired production of type I and III interferons in XLA patients following enteroviral infections (18), as well as dysregulated IL-1β signaling and NLRP3 inflammasome hyperactivation in BTK-deficient colitis models (19). Moreover, defective mucosal IgA responses have been implicated in impaired gut homeostasis and recognition of the intestinal microbiota in XLA (20). In our patient, enteropathy manifested early in childhood and progressed over two decades despite maximal supportive and immunosuppressive therapy. Although no additional pathogenic variants beyond *BTK* were detected, genetic testing was limited at the time of diagnosis to targeted Sanger sequencing. Histological findings of villous atrophy and absence of intestinal plasma cells, along with recurrent enteric infections—particularly norovirus and *Campylobacter jejuni*—are consistent with previously reported features of gastrointestinal involvement in humoral immunodeficiencies (6, 7). Notably, norovirus and *Campylobacter jejuni* were continuously detected in stool and mucosal biopsies and likely contributed to both immune activation and mucosal injury (8). Multiple empirical treatments—including enteral immunoglobulin, immunomodulators (azathioprine, sirolimus), and FMT—failed to achieve sustained improvement (21, 22). Ribavirin was discontinued early due to gastrointestinal intolerance, and nitazoxanide was not trialed due to poor evidence of efficacy in XLA (9). Targeted anti-cytokine therapies were not considered due to concurrent chronic infections with norovirus and *C. jejuni*, which contraindicate the use of systemic immunosuppression. Although prior data suggest that encapsulated FMT may be effective in chronic enteric infections (23), and case reports describe its successful use in CVID-associated enteropathy (24, 25) and in patients with hematologic malignancies (26), in our patient it led to significant clinical deterioration requiring hospitalization for gastroenteritis and sepsis—likely due to the introduction of live microorganisms in the context of a severely immunocompromised host and disrupted intestinal barrier. This therapeutic failure, coupled with severe malnutrition, osteopathy, and repeated hospitalizations, prompted consideration of HSCT as a salvage strategy.

The patient underwent myeloablative HSCT from a fully HLA-matched unrelated donor, with no early complications. Although conditioning regimens vary across the literature (1, 12), low-toxicity myeloablative protocols without total body irradiation—such as the one used here—appear effective. The favourable donor match and reduced-intensity regimen likely contributed to the absence of early adverse events and the excellent tolerability of the patient. A recent international survey (12) identified only 22 reported cases of HSCT in patients with XLA worldwide, predominantly in paediatric populations and primarily indicated for life-threatening infections or malignancies. The role of HSCT in adults, particularly for non-hematologic complications such as enteropathy, remains largely uncharted. To date, only one comparable case has been published (5), describing an 8-year-old patient who underwent HSCT for enteropathy associated with norovirus and *Campylobacter* jejuni coinfection. Our case contributes to the growing evidence that HSCT may be a safe and viable therapeutic option in selected adult patients, a group in which transplantation is often dismissed due to age and cumulative disease burden.

As in the case reported by Shillitoe et al. (5), our patient showed improvement in both the frequency and characteristics of stools within the first month post-transplant. *Campylobacter jejuni* was cleared during the first month, and norovirus by the second. This bacterial and virological clearance was paralleled by a marked improvement in humoral immune parameters, as evidenced by sustained increases in serum IgA, IgM, and IgG levels. Although total T-cell numbers remained comparable to pre-transplant levels, their renewed origin and restored BTK expression likely contributed to the clearance of both pathogens and the resolution of enteropathy through effective intestinal cellular immunity. Notably, endoscopic reassessment at 14 months demonstrated near-complete mucosal recovery, with histological evidence of B-cell and plasma cell reconstitution. These findings, together with sustained discontinuation of HPN and normalization of bone density, support the restoration of both mucosal integrity and broader systemic immune function.

Nearly two years post-HSCT, the patient has achieved partial B-cell reconstitution, characterized by a predominance of pre-germinal centre naïve B cells and limited numbers of class-switched memory B cells. Mild chronic GVHD developed but was effectively controlled with sirolimus and topical tacrolimus, without significant impact on quality of life. Notably, the patient remains on sirolimus, which may be contributing to delayed immune reconstitution. Sirolimus is a macrolide immunosuppressant that inhibits the mechanistic target of rapamycin (mTOR), a key regulator of cell growth, metabolism, and proliferation. By disrupting IL-2–mediated signal transduction, sirolimus selectively blocks the progression of antigen-stimulated T and B lymphocytes from the G1 to S phase of the cell cycle, impeding clonal expansion, memory cell formation, and antibody production (27, 28). This inhibitory effect on adaptive immune responses is likely to have contributed to the suboptimal serological response observed following administration of a *Salmonella typhi* Vi polysaccharide vaccine at 24 months post-transplant, while sirolimus therapy was ongoing. Moreover, the patient exhibited persistent mixed chimerism two years post-transplant, which may be contributing to a suboptimal response to neoantigens and the continued need for IgRT at last follow-up. It is also worth noting that successful immune control with stable mixed chimerism has been reported in the literature, including in patients with XLA who were ultimately able to discontinue IgRT (5, 29). Unlike in hematologic malignancies—where full donor chimerism is typically required to achieve cure—mixed chimerism can be sufficient for disease control in non-malignant conditions (30).

As so, in contrast to the cases reported by Shillitoe et al. (5) and Nishimura et al. (12), IgRT remains necessary in our patient, particularly to prevent recurrent respiratory infections, although the required dose has been substantially reduced. These findings are consistent with the notion that full immune reconstitution is a gradual process, potentially requiring a longer post-transplantation interval and complete withdrawal of immunosuppressive therapy. Nevertheless, even in the absence of complete IgRT discontinuation, HSCT may represent a more cost-effective long-term strategy compared to lifelong IgRT and prophylactic antibiotics. In high-income countries, the cumulative cost of IgRT remains substantial, and uninterrupted access cannot always be guaranteed, as highlighted during the COVID-19 pandemic (31). In resource-limited settings, where IgRT is often unavailable or unaffordable, HSCT may constitute the only viable therapeutic option, with post-transplant infectious burden effectively managed through antibiotic prophylaxis alone (32). Additionally, recent advances in gene editing will offer potential curative alternatives for XLA beyond HSCT. *In vitro* studies using base editing have successfully corrected BTK mutations in patient-derived hematopoietic stem cells, as demonstrated by Bahal et al. (33), restoring BTK protein expression and pre-B cell receptor signaling. Although clinical application remains under development, such strategies may transform future therapeutic approaches for XLA.

In conclusion, this case provides compelling evidence that allogeneic HSCT can be a safe, feasible, and potentially curative option in selected adult patients with XLA and severe, refractory non-hematologic complications such as enteropathy. While not yet considered standard of care, particularly in adult populations, our experience highlights the potential of HSCT to restore immune function, resolve chronic infection, and reverse long-standing gastrointestinal and nutritional morbidity. It also reinforces the need to reconsider earlier referral for HSCT in cases with associated organ damage and calls for prospective studies and long-term follow-up to better define its indications, outcomes, and cost-effectiveness.

## 4 Patient perspective

Since adolescence, the cumulative disease burden, chronic symptoms, and repeated hospitalizations had a profound psychosocial impact, progressively limiting the patient’s autonomy and social development. Psychiatric follow-up and pharmacological treatment were required for anxiety and depressive symptoms, particularly during periods of clinical worsening. One especially disabling episode occurred at 21 years of age, when he developed septic arthritis and osteomyelitis of the knee due to *C. jejuni*, which led to prolonged pain and significant difficulty in recovering joint mobility. This event further reduced his functional capacity and delayed his reintegration into educational and social activities. The combination of fatigue, nutritional dependence, chronic gastrointestinal symptoms, and mobility limitations significantly impaired his ability to pursue studies and maintain a normal lifestyle. These factors contributed to sustained emotional distress and a markedly reduced quality of life, necessitating ongoing multidisciplinary psychosocial support.

Following HSCT, the patient experienced a substantial improvement in his overall well-being. The resolution of chronic gastrointestinal symptoms and the withdrawal of parenteral nutrition significantly reduced his physical and emotional burden. With improved nutritional status, enhanced mobility, and fewer hospitalizations, he progressively regained autonomy and began re-engaging in social and educational activities that had long been out of reach. The stabilization of his health status also led to a marked reduction in anxiety and depressive symptoms, allowing for the gradual discontinuation of psychotropic medications under supervision. These improvements, both physical and psychological, were perceived by the patient and his family as transformative, restoring not only function but also hope and future perspective.

## Data Availability

The original contributions presented in the study are included in the article/supplementary material. Further inquiries can be directed to the corresponding author.
